# Rose Tree Club’s Resolutions on the Death of Dr. R. S. Huidekoper

**Published:** 1901-12

**Authors:** 


### RESOLUTIONS.

### ROSE TREE CLUB'S RESOLUTIONS ON THE DEATH OF DR. R. S. HUIDEKOPER.

THE following resolutions on the death of Dr. Rush Shippen Huidekoper were passed at the December meeting of the Rose Tree Fox Hunting Club:

"*Resolved*, That it is with the deepest regret and sorrow that we learn of the decease of our fellow-member, Dr. Rush Shippen Huidekoper, and mourn his taking off in the prime of life and in the full vigor of his manhood and business career. His long membership in this club endeared him to all connected with it, his generosity of character, genial disposition, and consideration for others winning for him the esteem and good-will of all who enjoyed a close friendship with him by which they could know his very many good and estimable qualities.

"This club owes much to his fondness for it and for the noble sport of fox-hunting, and his kindly traits, as well as energy, perseverance, and activity as a member, were largely instrumental in giving to the club its high standing for sociability, generosity in entertaining, and fearless riding in the field, among the lovers of fox-hunting all over the country, and largely aided in securing the good-will and respect of the farmers and land-owners of the county over whose domains the hunts extended.

"His early friendship with such well-known bold and fearless riders as Moncure Robinson, Jr., Dr. Daniel Bray, and Charles H. Townsend brought him into the club in 1875, when he was a student of medicine and surgery at the University of Pennsylvania and had just reached the years of manhood; and almost immediately he took an active and prominent part in its affairs, it being largely due to his activity and energy that the club races became an established feature in its sports, and that the club-house was erected and the club incorporated in 1881, and that gave to the club the increased and notable membership of that period of its existence and an enviable reputation over the country for high and honorable sportsmanship, and prominently coupled the name of Dr. Huidekoper and that of his favorite hunting mare, 'Pandora,' with it wherever and whenever the club was mentioned.

"His loss is deeply felt, and to his wife, who has been bereft of a kind, affectionate, and devoted husband, and to his family we tender our sincerest sympathy."

